# Glycogen synthase kinase 3β (GSK3β) and presenilin (PS) are key regulators of kinesin-1-mediated cargo motility within axons

**DOI:** 10.3389/fcell.2023.1202307

**Published:** 2023-06-09

**Authors:** Rupkatha Banerjee, Shermali Gunawardena

**Affiliations:** ^1^ Department of Neuroscience, The Herbert Wertheim UF Scripps Institute for Biomedical Innovation & Technology, Jupiter, FL, United States; ^2^ Department of Biological Sciences, The State University of New York at Buffalo, Buffalo, NY, United States

**Keywords:** kinesin-1, GSK3β, presenilin, axonal transport, phosphorylation

## Abstract

It has been a quarter century since the discovery that molecular motors are phosphorylated, but fundamental questions still remain as to how specific kinases contribute to particular motor functions, particularly *in vivo*, and to what extent these processes have been evolutionarily conserved. Such questions remain largely unanswered because there is no cohesive strategy to unravel the likely complex spatial and temporal mechanisms that control motility *in vivo*. Since diverse cargoes are transported simultaneously within cells and along narrow long neurons to maintain intracellular processes and cell viability, and disruptions in these processes can lead to cancer and neurodegeneration, there is a critical need to better understand how kinases regulate molecular motors. Here, we review our current understanding of how phosphorylation can control kinesin-1 motility and provide evidence for a novel regulatory mechanism that is governed by a specific kinase, glycogen synthase kinase 3β (GSK3β), and a scaffolding protein presenilin (PS).

## Introduction

Within axons, molecular motors transport essential components required for neuronal function, maintenance, and viability, and defects in axonal transport have been implicated in many neurodegenerative diseases including Alzheimer’s disease (AD). It is becoming increasingly evident that multiple levels of regulation must exist for the proper transport of a myriad of cargoes along axons, but to date, little is known about these mechanisms. We previously showed that Presenilin (PS), the catalytic component of γ-secretase which can also function as a scaffolding protein, and the kinase Glycogen Synthase Kinase 3β (GSK3β) can control the motility behaviors of amyloid precursor protein (APP)-containing vesicles under physiological conditions. Here we expand on predictions of our work and discuss how the scaffolding role for PS can bring or sequester not only GSK3β but other kinases to kinesin-1 containing vesicle complexes via its loop domain for phosphorylation/dephosphorylation switch mechanisms under physiological conditions.

### Regulation of kinesin-1 by phosphorylation

**FIGURE 1:**
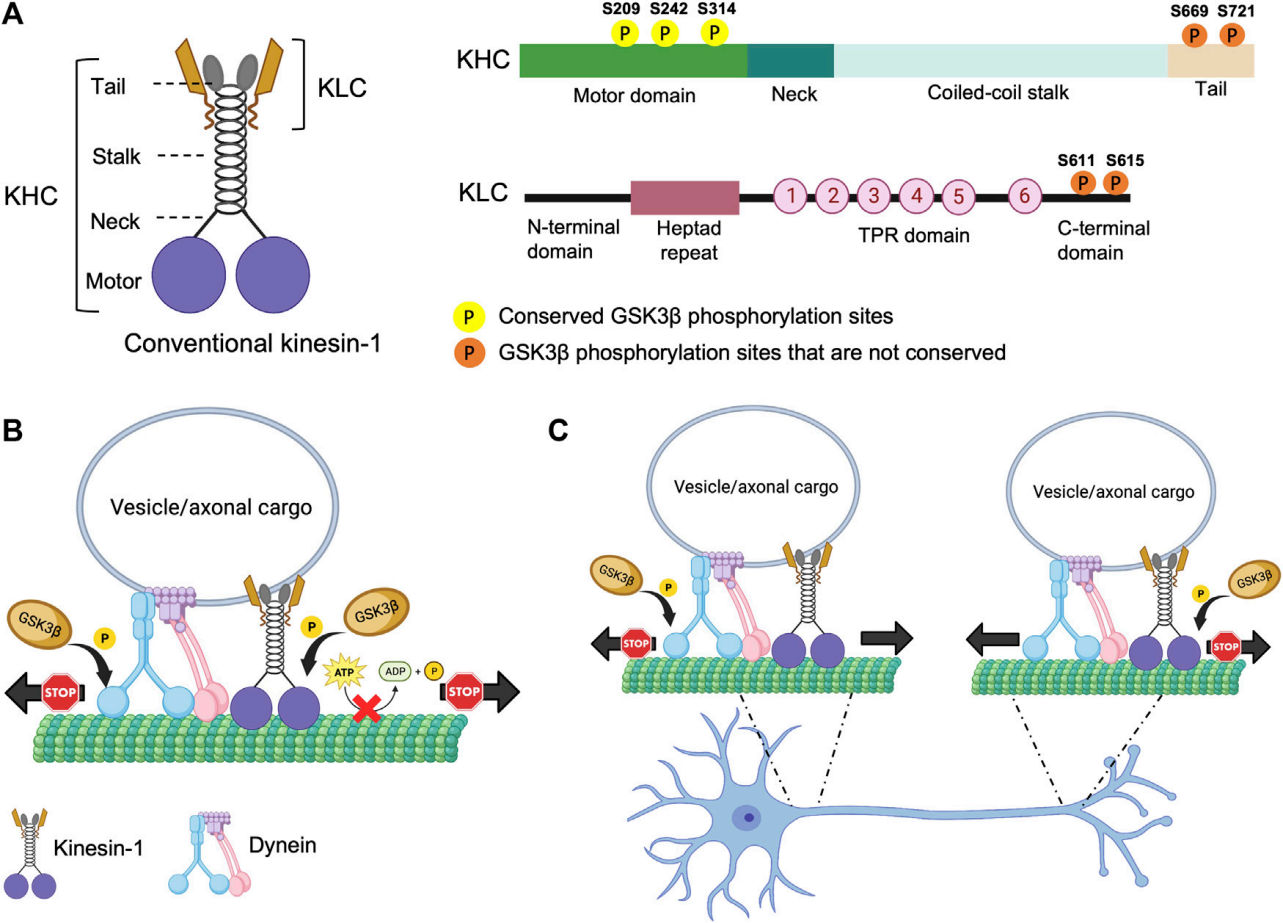
Structure of kinesin-1 and the putative phosphorylation sites. **(A)** *Drosophila* kinesin-1 is a homodimer consisting of KHC and KLC. KHC consists of a motor domain that contains the ATP and MT binding sites, a neck linker, a coiled-coil stalk, and a C-terminal tail domain. KLC consists of an N-terminal domain, a heptad repeat region that interacts with KHC, a highly conserved TPR region, and a C-terminal domain. **(B)** Differential phosphorylation of kinesin-1 and dynein by GSK3β may regulate motor coordination. **(C)** Under physiological conditions GSK3β-mediated phosphorylation/de-phosphorylation events of kinesin-1 and dynein motors at the axon initial segment (AIS) and/or at the distal axon could promote the directionally of cargo motility.

Phosphorylation/dephosphorylation of proteins, mediated by kinases and/or phosphatases is a widely utilized mechanism that orchestrates a vast array of cellular processes in a living organism. The anterograde molecular motor, kinesin-1, was identified as a phosphoprotein in 1995 by Lee and Hollenbeck (Lee and Hollenbeck, 1995). Later, a significant body of work suggested that phosphorylation likely governs the function of kinesin-1 during intracellular transport. Kinesin-1 is a heterotetrameric protein composed of two heavy chains (KHC) and two light chains (KLC) (Figure 1) (Bloom et al., 1988; Kuznetsov et al., 1988). KHC generates ATPase activity for anterograde motility, while KLC supports the attachment of cargoes to the kinesin-1 complex (Hirokawa et al., 2009). KHC has three structural domains (Figure 1A). The large globular N-terminal domain, also referred to as the motor domain contains the ATP and microtubule (MT) binding regions and is responsible for kinesin motor activity. The central alpha-helical coiled-coil domain is thought to mediate heavy chain dimerization. The small C-terminal tail domain interacts with KLC and other membranous organelles such as mitochondria. A new structural study has demonstrated that disruption of several associations between the motor, stalk and the tail domains of full length KHC is required for its activation (Tan et al., 2023). KLC has an N-terminal heptad repeat region that oligomerizes with the KHC coiled-coil, an acidic linker region, a tetratricopeptide repeat (TPR) region containing 6 TPR repeats (Gauger and Goldstein, 1993; Verhey et al., 1998; Wong and Rice, 2010; Zhu et al., 2012) and a C-terminal domain (Figure 1A). The TPR and the C-terminal regions are involved in cargo binding, functioning as a linker between KHC and its many cargos. The C-terminal domain can also regulate kinesin-1 activity (Amato et al., 2011). While the *Drosophila* genome contains only one gene each for KHC and KLC (Goldstein and Gunawardena, 2000), the mammalian genome is more complex containing three KHC genes (Kinesin-1A, B, C) and four KLC genes.

Early biochemical analysis implicated that protein kinase C (PKC) can phosphorylate both KHC and KLC (Matthies et al., 1993) while protein kinase A (PKA) can only phosphorylate KLC (Matthies et al., 1993). PKA-dependent KLC phosphorylation stimulated the ATPase activity of kinesin-1 (Matthies et al., 1993). Further, KLC phosphorylation by an unidentified kinase co-purified with the kinesin-1 holoenzyme was also able to increase MT-dependent ATPase activity and MT gliding *in vitro* (McIlvain et al., 1994; Lindesmith et al., 1997), suggesting that phosphorylation events are important for kinesin-1 function. Surprisingly, however, kinesin-1 lacks the target phosphorylation sites for PKC or PKA (Kumari and Ray, 2022) indicating that perhaps the PKC/PKA mediated effects observed are likely indirect or via phosphorylation events of accessory proteins. Since then, *in vitro* experiments have postulated that specific sites for c-Jun N-terminal kinase (JNK) and GSK3β exist on kinesin-1, and that these kinases are likely key regulators of kinesin-1 within neurons (Verhey et al., 1998; Morfini et al., 2002).

Sequence analysis suggests that mammalian kinesin-1 contains putative phosphorylation sites for several kinases including 5’ AMP-activated protein kinase (AMPK), casein kinase 2 (CK2), JNK (Hollenbeck, 1993; Morfini et al., 2009b; Schafer et al., 2009), and GSK3β (Banerjee et al., 2021), but the mechanistic significance of how diverse phosphorylation events by several kinases contribute to kinesin-1 function is not known. Since several phosphorylation sites for multiple kinases are located throughout the KHC motor, stalk and tail domains, and the KLC TPR and C-terminal domains, it is possible that different domains are controlled by phosphorylation switches to regulate and/or fine-tune kinesin-1 function. Further, some of the phosphorylation sites appear to be unique to specific KHC or KLC isoforms, while others are only partially conserved through evolution, demonstrating the evolutionary impact of phosphorylation events on motor function. Additionally, while it is intriguing that there are several phosphorylation sites on both KHC and KLC subunits (Table 1), perhaps not all sites get phosphorylated under physiological conditions.

**TABLE 1:** Summary of known kinesin-1 and adaptor protein phosphorylation sites across species.

Motor /Adapters	Subunit	Kinase	Residue	Physiological significance	Species	References
Kinesin-1	KHC	GSK3β	S314	Act as a stop for kinesin-1 motility, no effect on MT binding	*Drosophila melanogaster*	Banerjee et al. (2021)
		JNK	S175 (Kinesin-1B)	Stabilizies the folded conformation of kinesin and inhibits cargo and MT binding	*Mus musculus*	DeBerg et al. (2013), Padzik et al. (2016)
		JNK	S176 (Kinesin-1C)	Disengages kinesin-1 from MTs	*Mus musculus*	Padzik et al. (2016)
	KLC	PKA	unknown	Releases kinesin-1 from synaptic vesicles	*Rattus norvegicus*	Sato-Yoshitake et al. (1992)
		PKA	unknown	Stimulates ATPase activity	*Bos taurus*	Matthies et al. (1993)
		CAMKII	S240, S276	Facilitates transport of GLR-1, the AMPA-receptor subunit	*Caenorhabditis elegans*	Hoerndli et al. (2015)
		AMPK	S539, S575	Disrupts cargo binding	*Rattus norvegicus*	Amato et al. (2011)
		GSK3β	S615	Releases membrane-bound organelles	*Loligo pealii*	Morfini et al. (2002)
		CK2	unknown	Releases kinesin-1 from membranes	*Loligo pealii*	Pigino et al. (2009)
		ERK	S460	Weakens kinesin-1-Clstn1 interaction and inhibits Clstn1 mediated APP transport	*Rattus norvegicus*	Vagnoni et al. (2011)
		Unidentified	unknown	Increases MT-dependent ATPase activity and MT gliding in vitro	*Mus musculus*	Lindesmith et al. (1997), McIlvain et al. (1994)
JIP1/Aplip1	-	JNK	S421	Facilitates JIP1 interaction witth KHC and activates kinesin-1	*Mus musculus*	Fu and Holzbaur (2013)
		Wnd/MAPKKK, Hep/MAPKK	unknown	Inhibits JIP/Aplip1-KLC binding	*Drosophila melanogaster*	Horiuchi et al. (2007)
Alcα/Clstn1	-	CK1/CK2	multiple serines	Promotes kinesin-1-Alcα/Clstn1 interaction	*Mus musculus*	Sobu et al. (2017)
HTT	-	Akt	S421	Recruits kinesin-1 to BDNF vesicles and increases the anterograde motility of these vesicles	*Mus musculus*	Colin et al. (2008)
HAP	-	PKA	T598	Inhibits HAP1 association with KLC and prevents anterograde motility	*Rattus norvegicus*	Rong et al. (2006)

One functional significance for the phosphorylation-mediated switching events on kinesin-1 is to regulate motility by facilitating as well as inhibiting cargo binding. Indeed, early work showed that in mammalian cells, phosphorylation of KHC induced membrane association (Lee and Hollenbeck, 1995). KHC phosphorylation at serine 175 (S175) by JNK stabilized the folded conformation preventing cargo binding (Padzik et al., 2016), while PKA phosphorylation at an unknown site released kinesin-1 from synaptic vesicles (Sato-Yoshitake et al., 1992; DeBerg et al., 2013). In *C. elegans*, phosphorylation of S240 and S276 in the N-terminus of KLC2 by CaMKII augmented transport of the AMPA-receptor subunit, GLR-1 (Hoerndli et al., 2015; Hoerndli et al., 2022). In contrast, AMPK-mediated phosphorylation of the C-terminal domain of KLCs at S539 and S575 disrupted cargo binding (Amato et al., 2011), and phosphorylation at S615 by GSK3β released membrane-bound organelles (Morfini et al., 2002). Further, in squid axoplasm, activation of Casein Kinase 1 (CK2) by Aβ oligomers increased KLC phosphorylation causing kinesin-1 to be released from membranes (Pigino et al., 2009). Together, these observations speculate that perhaps phosphorylation of the C-terminus of KLC stabilizes the autoinhibited conformation of kinesin-1 which accounts for decreased affinity of the motors for cargoes, while phosphorylation of KHC or the N-terminus of KLC influences cargo transport by facilitating adaptor binding.

Adaptors are proteins that link molecular motors to cargoes, and adaptor phosphorylation is another probable mechanism for regulating motor recruitment and transport. For example, phosphorylation of the adaptor JIP-4 [(JNK) interacting protein-4] by JNK facilitates its interaction with the KHC tail and activates kinesin-1 *in vitro*, whereas dephosphorylated JIP1 binds to p150Dynactin, switching the movement of APP vesicles to the retrograde direction (Fu and Holzbaur, 2013). In other work, while phosphorylated adaptor protein Alcadeinα/Calsyntenin1 (Alcα/Clstn1) competes with JIP1 for KLC binding (Sobu et al., 2017), in rat cortical neurons phosphorylation of S460 on KLC1 by extracellular signal-regulated kinase (ERK) weakenes. kinesin-1-Alcα/Clstn1 interactions, thereby inhibiting Clstn1-mediated APP transport (Vagnoni et al., 2011). Further, phosphorylation of huntingtin (HTT) and huntingtin-associated proteins-1 (HAP1) on brain-derived neurotrophic factor (BDNF) containing vesicles by two competing kinases was proposed to coordinate the direction of motility. Akt-mediated phosphorylation of HTT at S421 can recruit kinesin-1 to BDNF vesicles increasing the anterograde motility of BDNF (Colin et al., 2008). Conversely, dephosphorylation of HTT causes kinesin-1 to be released from MT, promoting retrograde transport (Zala et al., 2008). Activation of retrograde movement of HTT can also occur via PKA-mediated phosphorylation of HAP1 at T598 which inhibits HAP1 association with KLC (Rong et al., 2006) preventing anterograde motility.

Phosphorylation events on the kinesin motor domain can also fine-tune kinesin-1 motor activity. We recently showed that GSK3β phosphorylation of KHC at S341 can act as a stop for kinesin-1 motility, with no effect on MT binding (Banerjee et al., 2021). In contrast, lack of phosphorylation at S341 resulted in uncoordinated motility with decreased attachment to MT and/or membranes, and reduced ATPase activity (Banerjee et al., 2021). Several other studies have also demonstrated a complex regulatory mechanism for phosphorylation events on S175 of the KHC motor domain. *In vitro* work using purified mammalian kinesin-1B showed that JNK-mediated phosphorylation at S175 decreased MT binding (DeBerg et al., 2013). Consistent with these findings, JNK phosphorylation of an equivalent S176 residue on mouse kinesin-1C disengaged 50% of motors from MTs (Padzik et al., 2016). Since the S175/176 residue is located in the loop8-β5 region of the kinesin motor domain that is involved in MT binding (Woehlke et al., 1997), perhaps increasing the negative charge in this loop alters the binding affinity of kinesin-1 to MTs without altering its ATPase activity. Therefore, perhaps JNK-mediated S175/176 phosphorylation on the KHC motor domain acts as a switch to stabilize the auto-inhibited conformation of kinesin-1 while increasing the minus-end-directed movement of cargo *in vivo*. The importance of S175 phosphorylation was further demonstrated under diseased conditions. In Huntington’s disease (HD), JNK3 activated by pathogenic HTT phosphorylated the conserved S175 in the motor domain of mouse kinesin-1A (Morfini et al., 2009a; Morfini et al., 2009b), inhibiting anterograde trafficking. Similarly, pathogenic superoxide dismutase (SOD) activated p38 MAP kinase to phosphorylate the same S175 residue, also inhibiting anterograde transport in squid axoplasm (Morfini et al., 2013). While it is unclear whether different kinases phosphorylate S175/176 on different classes of vesicles, it is evident that phosphorylation events on S175/176 of the kinesin motor domain are essential for the normal regulation of motor activity, and that these conserved phosphorylation events are also important in disease mechanisms. Therefore, specific phosphorylation events mediated by different kinases have distinct functional roles during kinesin-1 motility, demonstrating the complex mechanisms that likely exist to coordinate kinesin-1 activity during the transport of different cargos.

### The functional significance of GSK3β phosphorylation on kinesin-1

Several studies provide evidence to suggest that GSK3β can phosphorylate kinesin-1. The *Drosophila* KHC motor domain has three putative conserved GSK3β phosphorylation sites (Figure 1A). However, unlike mammalian KLC, *Drosophila* KLC lacks GSK3β phosphorylation consensus sequences (Banerjee et al., 2021). In cultured mammalian neurons, increased GSK3β activity increased KLC phosphorylation leading to decreased association of kinesin-1 to cargoes, while the ATPase activity or MT binding was unaffected (Morfini et al., 2002; Pigino et al., 2003). In contrast, in optical trap experiments in *Drosophila*, GSK3β activity influenced the number of active kinesin-1 motor complexes on cargoes/lipids (Weaver et al., 2013). In line with these observations, we previously showed that overexpression of constitutively active GSK3β increased the levels of kinesin-1 and dynein binding to cargoes (Dolma et al., 2014). But both the anterograde and retrograde synaptic vesicle velocities were decreased, indicating that GSK3β likely influences the activity of motors on vesicles. The discrepancy observed for GSK3β-mediated events on kinesin-1 in flies versus mice could be due to the fact that flies have only one KHC and KLC gene, while mammals have 3 genes each for KHC and KLC. Further, the 3 mammalian KHC genes (kinesin-1A, B, C) have diverse expression patterns in different tissues (Niclas et al., 1994; Nakagawa et al., 1997; Xia et al., 1998), with kinesin-1A and kinesin-1C expressed in neurons while kinesin-1B is ubiquitous. (Kang et al., 1999). Intriguingly, only kinesin-1A and kinesin-1B contain putative GSK3β phosphorylation sites, allowing us to speculate that specific phosphorylation events dictate functional specificity during cargo motility in different tissues. While these early studies suggest that GSK3β-mediated effects on motor function can be phosphorylation-dependent, the GSK3β target sites on kinesin-1 were not identified, and the precise molecular mechanisms by which GSK3β influenced motor function remains unclear.

We recently showed that GSK3β associates with and phosphorylates the *Drosophila* KHC motor domain at S314 (Banerjee et al., 2021). Our observations indicate that GSK3β-dependent phosphorylation act as a stop/go switch for kinesin-1 movement (Figure 1B). Constitutive GSK3β phosphorylation at S314 halts kinesin-1 motility without detaching the motor from MT. In contrast, disrupting GSK3β phosphorylation at S314 caused uncoordinated motility by decreasing MT and cargo binding, and reducing ATP hydrolysis. Disruption of GSK3β phosphorylation at S314 also led to impaired mitochondrial transport in *Drosophila* larval axons *in vivo* (Banerjee et al., 2021). The S314 residue resides in the α6 helix interfacing the head and the neck-linker domain. The neck-linker domain moves to a significant extent during the ATPase cycle to generate motor force along the MT, which likely increases tension transiently (Rice et al., 1999; Vale and Milligan, 2000) on the α6 segment during each stepping cycle (Qin et al., 2020). Therefore, there is a possibility that phosphorylation/dephosphorylation events at S314 by GSK3β could potentially alter the helix packaging and change the overall dynamics of how the neck-linker functions with the motor domain, which could likely contribute to the uncoordinated movement observed in the phospho-defective state.

An important unanswered question is how the activity of anterograde and retrograde motility is coordinated to achieve effective bi-directional movement of cargo *in vivo*. Under physiological conditions, most axonal cargoes and organelles are thought to contain both opposing motors bound at the same time (Maday and Holzbaur, 2012; Szpankowski et al., 2012), which are then activated/deactivated for regulated and coordinated motility (Barkus et al., 2008; Reis et al., 2012; Gunawardena S. et al., 2013; Lim et al., 2017). Both dynein and kinesin are phosphorylated by GSK3β. Work in mice showed that GSK3β can phosphorylate dynein intermediate chain (DIC), dynein light intermediate chains (DLICs), and dynein light chains (DLCs). Gao et al. (2015) However, the functional significance of these events is still elusive. Phosphorylation of DIC at S87/T88 by GSK3β reduced its interaction with the accessory protein Ndel1, which inhibited the retrograde movement of acidic organelles (Gao et al., 2015), suggesting that GSK3β phosphorylation of DIC can also act as a stop for dynein (Figure 1C), perhaps by affecting dynein force production. It is unknown whether GSK3β phosphorylation of DIC also influences anterograde motility. However, since the loss of GSK3β phosphorylation at KHC S314 affected both the anterograde and retrograde mitochondrial motility *in vivo* (Banerjee et al., 2021), we can speculate that differential GSK3β phosphorylation/de-phosphorylation events on motors presumably fine-tune and coordinate bi-directional motor activity under physiological conditions. In this context, since back-and-forth cargo motility is observed *in vivo* (Reis et al., 2012; Gunawardena S. et al., 2013; Weaver et al., 2013), perhaps defined GSK3β phosphorylation/de-phosphorylation events on kinesin/dynein coordinate and fine-tune the overall directionality of cargo movement. Alternatively, perhaps GSK3β-mediated phosphorylation/de-phosphorylation of kinesin-1 at the axon initial segment (AIS) facilitates anterograde movement, while dynein phosphorylation/de-phosphorylation by GSK3β at the distal axon promotes retrograde transport, with KHC phosphorylation at S314 acting as a stop (Banerjee et al., 2021) and DIC phosphorylation at S87/T88, decreasing dynein force generation by dissociating with Ndel (Gao et al., 2015) (Figure 1C). Further, site-specific GSK3β phosphorylation/de-phosphorylation events at cell bodies and/or at synapses could also facilitate cargo binding or cargo release from motors at the AIS or the distal axon.

### PS as a scaffolding protein for GSK3β and kinesin-1

PS moves bi-directionally within the peripheral nervous system (PNS) (Kasa et al., 2001; Papp et al., 2002) and central nervous system (CNS) axons (Shen et al., 1997). Several observations support the direct role of PS in the modulation of axonal transport (Stokin et al., 2008; Gunawardena S. et al., 2013; Dolma et al., 2014). PS was proposed to be present with APP containing axonal vesicles (Kamal et al., 2001). Consistent with this, sciatic nerve ligation experiments revealed that transgenic mice harboring two independent FAD-linked PS1 mutations exhibit severe impairment in the anterograde transport of APP and Trk receptors but not PrP (Lazarov et al., 2007). Genetic reduction of *Drosophila* PS stimulated the bi-directional velocities of APP vesicles, but not synaptotagmin (SYNT) vesicles (Gunawardena S. et al., 2013) indicating that PS selectively influences the trafficking of only a subset of kinesin-1-transported cargos. The transport defects induced by the loss of PS-mediated events on APP-vesicle movement could contribute to the defective neuronal and synaptic pathology observed in familial AD.

**FIGURE 2:**
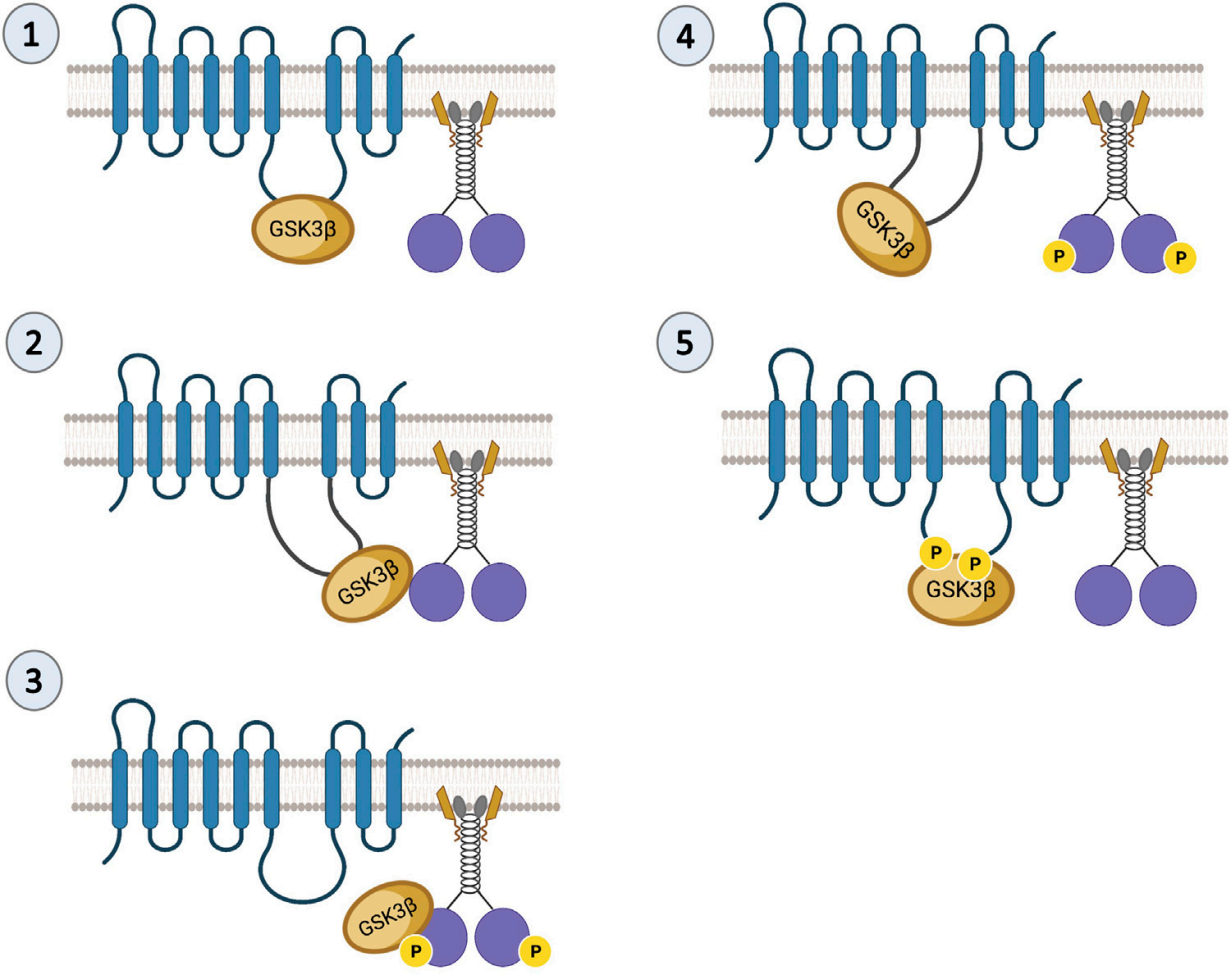
Predictions of the PS scaffolding model for GSK3β-mediated functions on kinesin-1. In steps 1 and 2 the PS loop can act as a scaffold to bring GSK3β to kinesin-1 during cargo motility. In step 3, once GSK3β and kinesin-1 associate, GSK3β can phosphorylate kinesin-1. In step 4 once GSK3β phosphorylates kinesin-1, the PS loop can sequester GSK3β away from kinesin-1. In step 5, phosphorylation of the PS loop by GSK3β can facilitate the PS-GSK3β interaction to prevent GSK3β from acting on motors.

A similar phenotype to the reduction of *Drosophila* PS on APP motility was observed for the reduction of *Drosophila* GSK3β (Weaver et al., 2013), suggesting that PS and GSK3β are functionally coupled during APP transport. Indeed, several studies indicate that PS is an unprimed substrate for GSK3β (Takashima et al., 1998; Prager et al., 2007; Uemura et al., 2007). PS and GSK3β biochemically associate with each other (Takashima et al., 1998; Kang et al., 1999), and functional interactions between PS and GSK3β have been reported during axonal transport (Dolma et al., 2014). PS or GSK3β loss-of-function mutants are lethal. Larvae homozygous for PS or GSK3β loss-of-function mutations demonstrate paralytic crawling phenotypes and do not eclose to adults (Dolma et al., 2014). Both PS or GSK3β mutant larvae showed disrupted transport with axonal blockages (Weaver et al., 2013; Dolma et al., 2014) at levels comparable to the homozygous loss-of-function motor protein mutants (Hurd and Saxton, 1996). Intriguingly, loss-of-function PS or GSK3β mutants showed decreased levels of active GSK3β together with decreased kinesin-1 and dynein binding to membranes (Dolma et al., 2014). Together, these observations establish an essential role for both PS and GSK3β during axonal transport. Work in cells suggested that GSK3β can phosphorylate KLC, releasing kinesin from vesicles (Morfini et al., 2002), and work in flies showed that GSK3β phosphorylates KHC at S314 causing kinesin motors to stop while still bound to MT (Banerjee et al., 2021). It is possible that PS plays a scaffolding role in controlling GSK3β-mediated roles on kinesin-1 subunits during axonal transport. Since the hydrophilic loop region of PS binds GSK3β (Takashima et al., 1998), the PS loop could either bring GSK3β to motors (Figure 2, Step 1–3) or sequester GSK3β away from motors (Figure 2, Step 4–5). Indeed, deletion of the PS loop region caused axonal transport defects while overexpressing the PS loop had no effect. Therefore, we proposed that the hydrophilic PS loop region likely sequesters GSK3β away from kinesin-1 to rescue axonal transport defects mediated by excess GSK3β (Banerjee et al., 2018).

**FIGURE 3:**
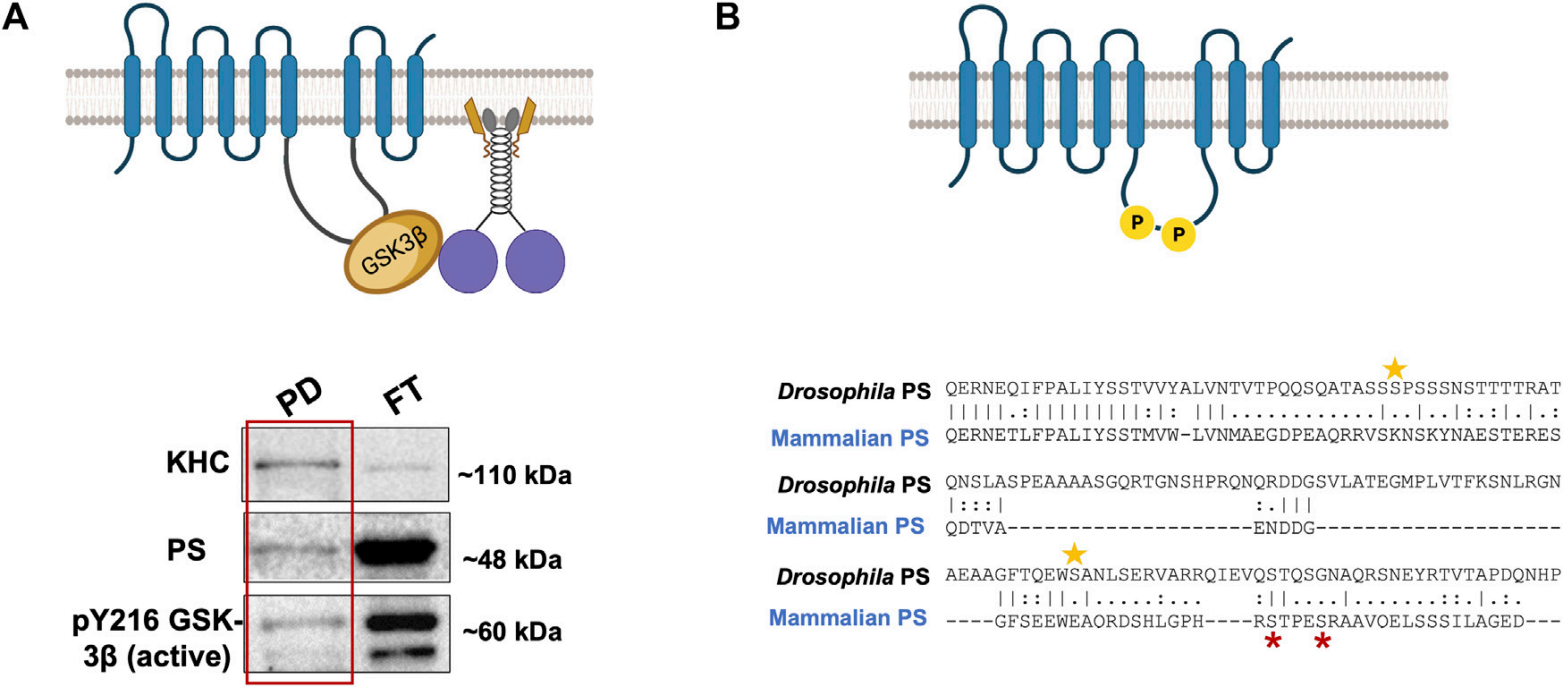
**(A)** PS-GSK3β-kinesin-1 form a complex. A schematic diagram of PS, GSK3β, and kinesin-1 interactions during cargo motility. Western blot analysis show PS and active GSK3β are present with KHC in the pull down (PD) fraction from a *Drosophila* KHC immunoprecipitation assay, suggesting PS-GSK3β-kinesin-1 likely associate. FT- flow through (Banerjee, 2020). **(B)** The *Drosophila* PS loop has 2 putative GSK3β phosphorylation sites. Two putative sites on serine 333 and 408 (yellow stars) are present on the *Drosophila* PS loop. GSK3β is known to phosphorylate the human PS loop at serine 353 and 357 (red stars). The sequence alignment indicates that mammalian and fly GSK3β phosphorylation sites are not evolutionarily conserved.

There are at least two predictions for the PS scaffolding model for GSK3β-mediated functions on kinesin-1. One prediction is that since GSK3β and kinesin-1 associate with each other (Morfini et al., 2002; Banerjee et al., 2018), and PS associates with GSK3β via the loop (Marfany et al., 1998; Ye and Fortini, 1998; Wolfe et al., 1999; Steiner and Haass, 2000), then PS, GSK3β, and kinesin-1 should also be associated together to form a complex. Indeed, we have successfully pulled down the human PS loop (hPSloop) and active-GSK3β with *Drosophila* KHC (Figure 3A; Banerjee, 2020). However, further study is needed to determine where the PS-GSK3β-kinesin-1 complex is localized and whether the scaffolding role of PS takes place in the cell bodies or within axons.

**FIGURE 4:**
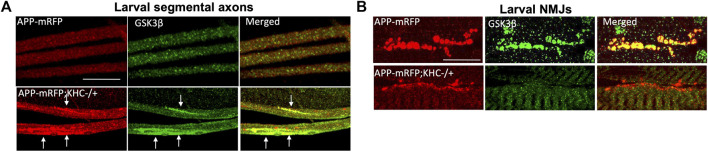
Reduction of KHC disrupts the co-localization of APP and GSK3β during axonal transport. **(A)** Heterozygous reduction of KHC (KHC−/+) show APP (red) and GSK3β (green) containing axonal blocks (arrows). **(B)** APP and GSK3β are co-localized within larval NMJs indicating that both are transported together. Reduction of KHC decreases both APP and GSK3β within NMJs compared to WT (Gunawardena et al., 2013b). Scale bar = 10 μm.

Since PS is a membrane-bound protein, the second prediction is that the PS scaffolding function occurs on membranes. Indeed, endogenous PS1 localizes at the plasma membrane as a biologically active molecule (Uemura et al., 2007). While excess PS/PS loop increased active GSK3β and kinesin-1 binding to membranes and rescued GSK3β-mediated axonal blockages, genetic reduction of PS led to axonal blockages by decreasing both active GSK3β and kinesin-1 binding to membranes (Dolma et al., 2014; Banerjee et al., 2018). Further, PS and active GSK3β are present in larval cell bodies, axons, and NMJs (Banerjee et al., 2018; Figure 4; Gunawardena SD. et al., 2013), and reduction of KHC (Figure 4; Gunawardena SD. et al., 2013) or loss of GSK3β activity (Banerjee et al., 2018) disrupted GSK3β localization to the NMJs, suggesting that PS, active GSK3β and KHC are likely present together on synaptic vesicles. GSK3β-mediated kinesin-1 binding to membranes is probably a step-by-step process that is mediated by PS. Initially, PS brings GSK3β to motors to allow GSK3β to associate with motors (Morfini et al., 2002; Banerjee et al., 2018). The GSK3β-motor association likely facilitates motor phosphorylation (Banerjee et al., 2018; Banerjee et al., 2021) (Figure 2, Step 1–3). Once the motor is phosphorylated, PS may sequester GSK3β away from motors (Figure 2, Step 4). Therefore, GSK3β-mediated phosphorylation/dephosphorylation events can direct motor attachment/detachment to membranes and motor activity.

Alternatively, phosphorylation of PS could act as a molecular switch that turns off the GSK3β-mediated effects on motor activity or motor-cargo binding. In the Wnt-β-catenin pathway, GSK3β phosphorylates the PS loop at serine residues 353 and 357 (Twomey and McCarthy, 2006; Prager et al., 2007; Uemura et al., 2007), and phosphorylation induces structural changes in the PS loop reducing GSK3β-β-catenin interaction, decreasing β-catenin phosphorylation and degradation (Prager et al., 2007). Further, GSK3β activity modified the localization and function of PS (Uemura et al., 2007; Banerjee et al., 2018). GSK3β-phosphorylation-mediated conformational changes in the loop could facilitate GSK3β-PS interactions preventing GSK3β-kinesin-1 association, thus turning off GSK3β-mediated effects on motors (Figure 2, Step 5). While the mammalian PS loop has two documented GSK3β phosphorylation sites (Prager et al., 2007), sequence analysis show that the *Drosophila* PS loop region also has 2 putative GSK3β phosphorylation sites, two serines at positions 333 and 408 (Figure 3B). However, these residues are not conserved. *Drosophila* PS is about 50% identical to human PS-1 and PS-2 at the amino acid sequence level, and the ∼30 most amino-terminal residues of the loop region share high homology with the human PS1 loop. Further, a 14-amino-acid alternative splice variant in the loop domain generates two PS isoforms in *Drosophila* (Ye and Fortini, 1998). Regardless of these differences, binding of proteins to the PS loop either directly (for, e.g., Filamin, (Guo et al., 2000)) or via the aid of co-factors (for, e.g., β- and δ-Catenin, (Noll et al., 2000)) together with the essential PS-meditated roles in neuronal protection during aging, appear to be evolutionarily conserved between flies and mammals (Kang et al., 2017). Consistent with this, both *Drosophila* and human PS loop regions can rescue active GSK3β-mediated axonal blockages (Banerjee et al., 2018). While it is still unknown whether S333 and S408 in the *Drosophila* PS loop are phosphorylated, and whether phosphorylation of the PS loop negatively affects GSK3β-mediated events on kinesin-1, further study would be needed to isolate how phosphorylation of PS contributes to its scaffolding role.

### The physiological relevance of the PS-GSK3β-kinesin-1 scaffold model during cargo motility

**FIGURE 5:**
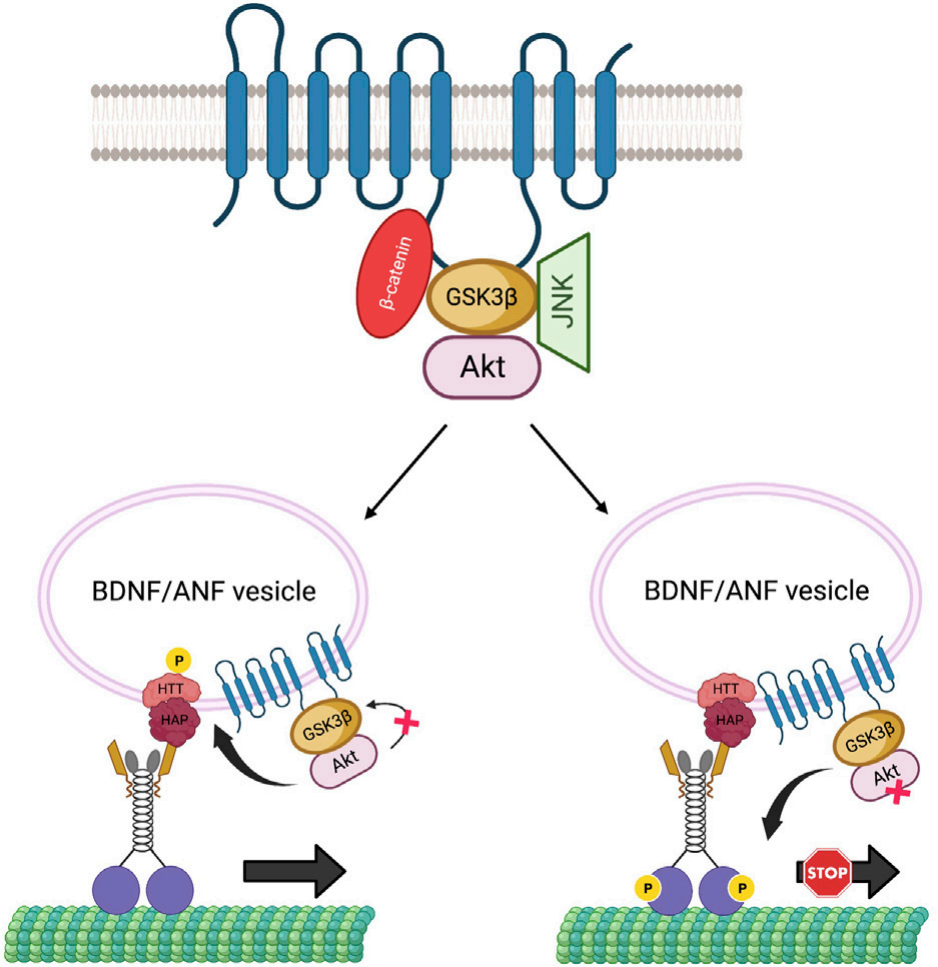
The PS scaffolding model for the regulation of BDNF/ANF vesicle motility within axons. The PS loop can associate with Akt, GSK3β and JNK in a large complex to regulate Akt, GSK3β, JNK activities during cargo motility. Activation of Akt can inactivate GSK3β to facilitate the anterograde movement of BDNF/ANF vesicles by HTT phosphorylation and recruitment of kinesin-1, while activation of GSK3β by PS can act as a stop for BDNF/ANF vesicles.

Growing evidence hints at the role of scaffolding proteins in regulating cargo-specific motility (Kural et al., 2005). PS can act as a scaffold to bring GSK3β and β-catenin via the hydrophilic loop in the Wnt-β-catenin pathway (Murayama et al., 1998; Takashima et al., 1998), and FAD-linked PS mutations or reduction of PS impacts GSK3β activity (Lazarov et al., 2007; Dolma et al., 2014; Banerjee et al., 2018), indicating that PS plays a key role in modulating GSK3β functions. It is also possible that other regulatory kinases that influence axonal transport are a part of the PS scaffolding complex. For example, PS may also affect Akt functions. Indeed, in cultured hippocampal neurons, FAD-mutant PS induced apoptosis, by downregulating Akt kinase activity (Weihl et al., 1999). Interestingly, Akt can interact with GSK3β and phosphorylate GSK3β on serine 9 to inactivate GSK3β (van Weeren et al., 1998), implying that perhaps Akt, GSK3β, and PS can form a complex. Further, Akt influences the axonal movement of BDNF-containing vesicles by phosphorylating the scaffolding protein HTT (Colin et al., 2008), while GSK3β influences the movement of a wide range of cargoes including APP, synaptobrevin (syb), neuropeptide ANF and mitochondria (Banerjee et al., 2021; Dolma et al., 2014; Weaver et al., 2013, (Dolma et al., 2014; Iacobucci and Gunawardena, 2018). Thus, it is possible that PS associates with GSK3β, β-catenin, and/or Akt in a large complex via the loop region, to function as a regulatory unit that controls GSK3β and Akt-specific activities. In this scenario, activation of Akt by PS would inactivate GSK3β, and phosphorylate HTT to recruit kinesin-1 to BDNF/ANF-containing vesicles for anterograde motility. Conversely, activation of GSK3β by PS would trigger kinesin-1 phosphorylation to regulate APP, syb containing vesicles or mitochondria motility (Figure 5). Since GSK3β activity also influences the motility of neuropeptide (ANF) containing dense core vesicles (Dolma et al., 2014; Iacobucci and Gunawardena, 2018), and most ANF-containing vesicles also contain BDNF (Xia et al., 2009), GSK3β-mediated phosphorylation may also regulate BDNF motility via kinesin-1 phosphorylation. Additionally, a recent study in mouse primary neurons showed that loss of PS1 function caused JNK activation which hyperphosphorylated dynein intermediate chain (DIC), leading to impaired retrograde transport of endosomes (Lie et al., 2022). The PS scaffolding complex could therefore contain several kinases including GSK3β, Akt, and JNK (Figure 5). In this scenario, the PS loop may synchronize the functions of these kinases under physiological conditions to mediate specific kinesin-1 and dynein motor activities for fine-tuning bi-directional movement of several different cargos along axons.

**FIGURE 6:**
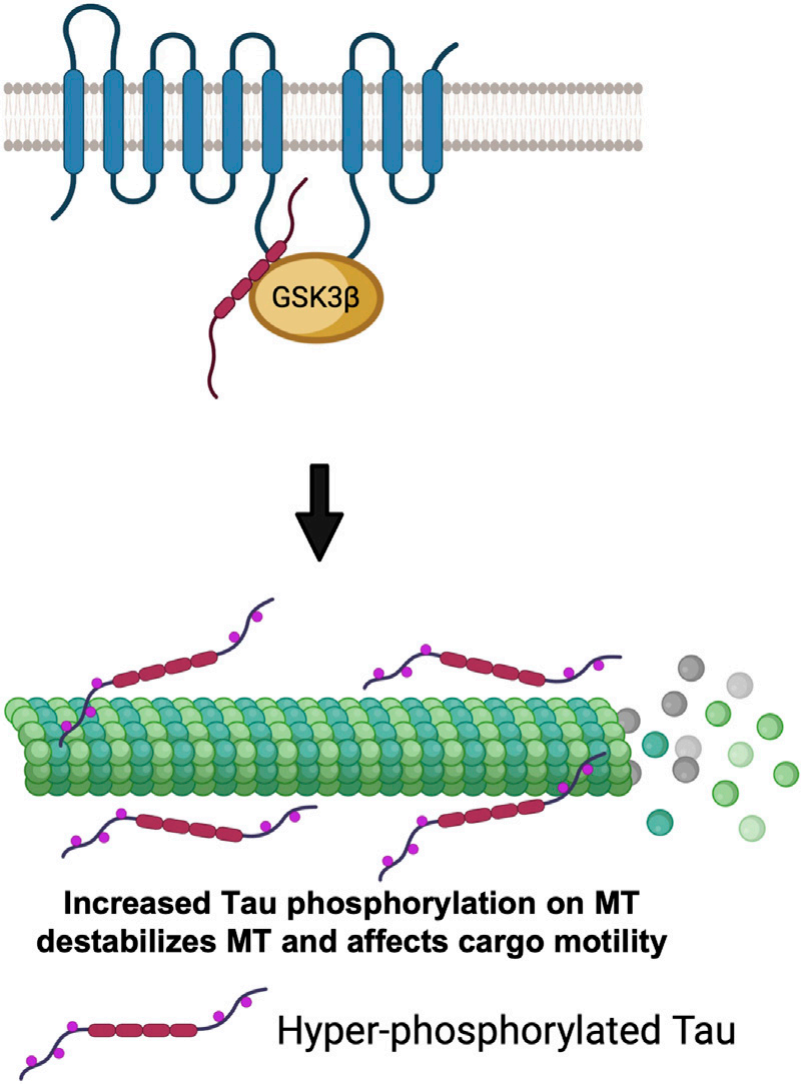
The PS scaffolding model for the regulation of cargo motility via GSK3β-mediated phosphorylation of Tau. PS loop can regulate GSK3β mediated Tau phosphorylation. Hyper-phosphorylated Tau detaches from MTs, destabilizes MTs and impairs cargo motility.

Since GSK3β is known to phosphorylate Tau (Ali et al., 2001), and excess active-GSK3β enhanced Tau-dependent transport defects in *Drosophila* motor neurons (Mudher et al., 2004), an alternative possibility is that the PS-GSK3β scaffold could also regulate kinesin-1 motility by influencing the MT-associated protein Tau. While studies have shown that the amount of Tau associated with MT can influence kinesin-1 and dynein motility (Dixit et al., 2008), it is unclear whether the observed transport defects are due to altered motor binding to MTs or disruption of MT tracks. Intriguingly, however it was reported that both Tau and GSK3β can bind to the same region (residues 250–298) on the PS loop (Takashima et al., 1998). Loss of PS (Soto-Faguas et al., 2021) and FAD-mutations in the PS loop (Takashima et al., 1998) also increased Tau phosphorylation. Hyper-phosphorylated Tau dissociated from MTs causing axonal transport defects (Pigino et al., 2001). Therefore, although speculative, perhaps the PS loop could act as a molecular tether to connect GSK3β to its substrate Tau to regulate Tau function by phosphorylation. In this context, perhaps PS-mediated changes in the GSK3β phosphorylation state of Tau could govern the amount of Tau associated with MTs (Figure 6). Moreover, it is also possible that PS-mediated GSK3β phosphorylation of Tau could contribute to Tau degradation. Further studies will be needed to test predictions of this proposal.

### PS-GSK3β-kinesin-1 scaffold model in the axonal transport of APP and its implication for AD

Over 150 mutations in PS have been implicated in familial AD (FAD) (Takashima et al., 1998; Gantier et al., 2000). FAD mutations alter toxic Aβ peptide fragments, which are generated by sequential proteolytic cleavage of APP by β-secretase followed by γ-secretase (Scheuner et al., 1996; Xia et al., 2015; Sun et al., 2017). While Aβ40 is the most abundant form of the Aβ peptide, Aβ42 is considered to be the pathogenic, toxic form that forms plaques. Studies have shown that FAD PS mutations increase the proportion of Aβ42 (Borchelt et al., 1996; De Strooper et al., 1998; Selkoe, 1998), however, it is unclear whether increases in Aβ42 is due to a gain-of-function (De Strooper et al., 1998; Woodruff et al., 2013) or a loss-of-function mechanism (Saura et al., 2004; Kelleher and Shen, 2017). Loss of PS function causes memory loss, synaptic plasticity defects, and age-dependent neurodegeneration (Saura et al., 2004). PS deficiency can result in inflammatory responses with an accumulation of pathological Tau in neuronal and glial cells, indicating roles for PS in neurofilament assembly and neurite extension (Dowjat et al., 2001; Pigino et al., 2001; Soto-Faguas et al., 2021). PS dysfunction is also associated with Tau pathology independent of Aβ with PS mutations linked to Tau aggregation in the frontal cortex in frontotemporal dementia (FTD) (Raux et al., 2000; Dermaut et al., 2004).

GSK3β is another major player in AD pathogenesis (Hooper et al., 2008). Studies show that GSK3β activity and/or protein levels are elevated in the brains of AD patients (Pei et al., 1997; Leroy et al., 2007). In various cell culture systems, and in invertebrate and mammalian models of AD, increased GSK3β activity led to hyper-phosphorylation of Tau (Hanger et al., 1992; Ishiguro, 1998). Hyper-phosphorylated Tau resulted in the formation of insoluble neurofibrillary tangles (NFTs), the other pathological hallmark of AD (Neddens et al., 2018), implying a role for GSK3β mediated phosphorylation in the formation of NFTs. Further, increased GSK3β activity was proposed to increase Aβ generation (Zhao et al., 2004). Therefore, given the pivotal roles of PS and GSK3β in AD, it is possible that the axonal transport defects mediated by the loss of PS-mediated regulatory effects on GSK3β contribute to AD pathogenesis.

**FIGURE 7:**
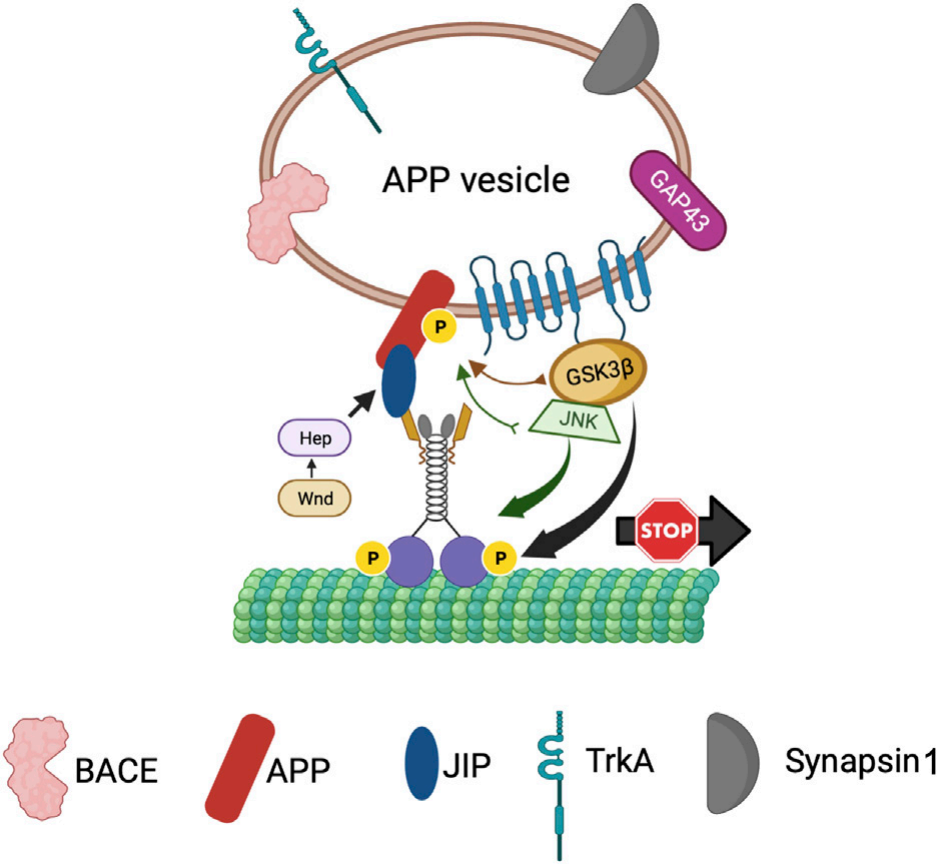
The PS scaffolding model for the regulation of APP vesicle motility within axons. PS is present within APP containing vesicles together with BACE, TrkA, Synapsin1 and GAP43. The PS loop associates with GSK3β and JNK to bring GSK3β/JNK to APP perhaps for phosphorylation of APP to control the directionality of APP vesicle motility, while activation of GSK3β by PS can act as a stop for APP vesicles.

Previous work proposed that axonal transport dysfunction caused by faulty transport of APP by kinesin-1 is a critical event in AD progression (Stokin et al., 2005; Stokin and Goldstein, 2006). Both APP and PS are transported bi-directionally within axons. PS was shown to move bi-directionally in rat sciatic nerves (Kasa et al., 2001; Papp et al., 2002) and from the entorhinal cortex to the hippocampus via axons of the perforant pathway (Sheng et al., 2003). APP is transported within a subclass of vesicles that contain PS and BACE, the two secretases necessary for the cleavage of APP (Kamal et al., 2001). Further, using double-ligation experiments Kamal et al. demonstrated that Aβ42 can be generated within axons as all components necessary for APP cleavage were onboard APP vesicles transported by kinesin-1. Overexpression of human wild-type APP or FAD-linked APP mutations attenuated axonal transport in both *Drosophila* and mice (Gunawardena and Goldstein, 2001; Stokin et al., 2005), suggesting that excess APP or aberrant APP processing can lead to transport defects by sequestering motors. These results together with the observation that the C-terminal region of APP can interact with KLC directly (Kamal et al., 2000; Satpute-Krishnan et al., 2006) or indirectly via JIP1 (Inomata et al., 2003) for anterograde motility (Figure 7) supports the hypothesis that excess APP causes axonal transport defects by interacting with and titrating kinesin-1 away from the soluble pool (Gunawardena and Goldstein, 2001). Interestingly, excess PS or excess PS loop rescued APP-induced axonal transport defects (Stokin et al., 2008). Since the PS/PS loop-mediated rescue of APP-mediated transport defects is akin to the PS-dependent suppression of GSK3β-mediated blockages (Banerjee et al., 2018), perhaps the same mechanism accounts for the rescue of transport deficits. Indeed, loss of PS or GSK3β function resulted in an identical phenotype where APP vesicle velocities were stimulated (Gunawardena S. et al., 2013; Weaver et al., 2013; Iacobucci et al., 2018). Taken together, these observations hint that PS and GSK3β are likely key players in the regulation of APP motility within axons. Based on the PS scaffolding model, we can speculate that the hydrophilic PS loop regulates GSK3β-mediated kinesin-1 functions on APP vesicles (Figure 7). However, work has shown that APP is phosphorylated at Thr668, and phosphorylation of APP can regulate the formation of an APP-JIP1 complex (Muresan and Muresan, 2005) to possibly function as a molecular switch to control the directionality of APP motility (Fu and Holzbaur, 2014). Therefore, perhaps the PS loop can also play a role in mediating APP phosphorylation. Intriguingly, MAPKKK Wnd and its downstream MAPKK Hep were shown to regulate the attachment of the APP-JIP1 cargo linker to kinesin-1 (Horiuchi et al., 2007). Although further investigation is required, these observations strengthen the scaffolding model for PS whereby the PS loop likely coordinates the functions of several kinases to guide the bi-directional axonal movement of APP vesicles under physiological conditions.

In AD brains, both GSK3β activity and the levels of phosphorylated APP are upregulated (Chang et al., 2006), implying a role for GSK3β in APP phosphorylation. APP can be phosphorylated at T668 by GSK3β (Aplin et al., 1996) in addition to Cdk5 (Iijima et al., 2000), Cdk2 (Suzuki et al., 1994), or JNK (Standen et al., 2001; Kimberly et al., 2005). Neuron-specific phosphorylation of APP at T668 is important for the axonal transport of a sub-class of APP that is phosphorylated and bound to JIP1 which is moved by associations with kinesin-1 and accumulates at growth cones (Muresan and Muresan, 2005). T668 phosphorylation facilitates APP cleavage by BACE leading to increased Aβ generation (Lee et al., 2003). Further, in AD patients, NF-κB is overexpressed and mediates GSK3β-induced BACE-1 expression (Chen et al., 2012), which likely contributes to increased Aβ generation. Indeed, increased Aβ can block Wnt-mediated GSK3β-inhibition leading to further increases in Aβ formation and Tau hyperphosphorylation (Magdesian et al., 2008). We can hence postulate that GSK3β may aid APP-KLC associations perhaps by phosphorylating APP at T668. In this context, PS may act as a negative regulator of APP-KLC interaction by titrating GSK3β away and preventing APP phosphorylation (Figure 7). Unphosphorylated APP will no longer associate with kinesin-1, and kinesin-1 will be released from APP vesicles leading to an increased pool of kinesin-1 available for axonal transport. An enhanced supply of kinesin-1 motor could rescue the APP-induced axonal accumulations. Additionally, GSK3β could also influence the subcellular localization of APP. Consistent with this hypothesis, the reduction of kinesin-1 caused APP and GSK3β containing axonal blockages and decreased APP-GSK3β localization to the neuromuscular junctions (NMJs) (Figure 4; Gunawardena SD. et al., 2013). The PS-mediated rescue of APP-induced transport defects (Stokin et al., 2008) could be due to the restriction of APP to cell bodies, preventing APP entry into axons via associations with kinesin-1. Indeed, this model is consistent with our previous work that proposed that PS reduction enhanced the sorting of APP from the cell body to the axon (Gunawardena S. et al., 2013). According to this model, disruption of the PS loop would prevent PS-APP-GSK3β complex formation, and APP would no longer be restricted to the cell bodies. Increased APP in axons would bind to and titrate kinesin-1 away from non-APP vesicles resulting in axonal accumulations of non-APP vesicles (Gunawardena and Goldstein, 2001). While APP vesicles contain both kinesin-1 and dynein (Szpankowski et al., 2012), whether PS loop-mediated events modulate dynein-mediated APP transport remains undetermined. Further study would be needed to test the predictions proposed here and to isolate how a myriad of kinases contribute to regulatory switch mechanisms during cargo motility.

## Conclusion

In this review, we discuss how GSK3β and PS play critical roles in regulating kinesin-1-mediated cargo motility within axons. We provide evidence for a scaffolding role for PS in sequestering or bringing GSK3β and perhaps additional kinases to kinesin-1 containing vesicle complexes via its loop domain for phosphorylation/dephosphorylation switch mechanisms. While we propose that these events can occur on APP vesicles and perhaps on BDNF-HTT vesicles, whether similar mechanisms govern the motility of other vesicle types is unknown. Furthermore, while it is likely that PS functions as a molecular tether for several regulatory proteins, whether PS is involved in the regulation of dynein motors which are also phosphorylated by GSK3β is unclear and warrants future investigation.

## Figures and Tables

**Table:** 

**Aβ**	Amyloid beta
**AD**	Alzheimer’s disease
**APP**	Amyloid Precursor Protein
**AKT**	*AKT* serine/threonine *kinase* 1. Also known as Protein *kinase*B (PKB)
**Alcnα**	Alcadeinα
**AMPK**	Adenosine Monophosphate-activated protein kinase
**ATPase**	Adenoisne Triphosphatase
**BACE**	Beta-secretase
**BDNF**	Brain-derived neurotrophic factor
**CaMKII**	Calmodulin dependent protein kinase II
**Cdk**	Cyclin dependent kinase
**CK2**	Casein Kinase 2
**Clstn1**	Calsyntenin1
**CNS**	Central nervous system
**DIC**	Dynein intermediate chain
**DLC**	Dynein light chain
**DLIC**	Dynein light intermediate chain
**ERK**	Extracellular signal-regulated kinase
**FAD**	Familial Alzheimer’s Disease
**FTD**	Frontotemporal dementia
**GSK3β**	Glycogen synthase kinase 3β
**HD**	Huntington’s disease
**HTT**	Huntingtin
**JIP**	JNK-interacting protein
**JNK**	Jun N-terminal kinase
**KHC**	Kinesin heavy chain
**KLC**	Kinesin light chain
**MAPK**	Mitogen activated protein kinase
**MT**	Microtubule
**NFTs**	Neurofibrilary tangles
**PKA**	Protein kinase A
**PKC**	Protein kinase C
**PrP**	Prion protein
**PS**	Presenilin
**SOD**	Superoxide dismutase
**TPR**	Tetratricopeotide repeat
